# Macronutrient balance and micronutrient amounts through growth and development

**DOI:** 10.1186/s13052-021-01061-0

**Published:** 2021-05-08

**Authors:** Giovanni Savarino, Antonio Corsello, Giovanni Corsello

**Affiliations:** 1 Department of Health Promotion, Mother and Child Care, Internal Medicine and Medical Specialties “G. D’Alessandro”, University Hospital “P.Giaccone”, Palermo, Italy; 2 Pediatric Unit, Fondazione IRCCS Ca’ Granda Ospedale Maggiore Policlinico, Milan, Italy; 3 University of Milan, Milan, Italy

**Keywords:** Nutrition, Pediatric growth, Vitamins, Macronutrients, Micronutrients, Trace elements, Zinc, Iron, Vitamin D, Breast milk, Infant nutrient intakes

## Abstract

Nutrition is essential for human growth, particularly in newborns and children. An optimal growth needs a correct diet, in order to ensure an adequate intake of macronutrients and micronutrients. Macronutrients are the compounds that humans consume in largest quantities, mainly classified in carbohydrates, proteins and fats. Micronutrients are instead introduced in small quantities, but they are required for an adequate growth in the pediatric age, especially zinc, iron, vitamin D and folic acid. In this manuscript we describe the most important macro and micronutrients for children’s growth.

## Introduction

Nutritional science principally distinguishes two different classes among its classifications: macronutrients and micronutrients [1]. Macronutrients can be considered as the main components of different tissues, and they constitute the total amount of the caloric intake, meaning the principal energy source of the human body; they are mainly distinguished in carbohydrates, proteins and lipids [2]. Micronutrients are instead those components of diet that do not provide a significant contribution on the caloric intake, but can still be considered crucial for health and vital functions, even if needed in smaller amounts. They principally include vitamins (both fat-soluble and water-soluble) and minerals [3].

Since both macro and micronutrients’ deficiencies can have a significant impact on the whole function and physiology of the human body, particularly on the various processes of growth, a step-by-step distinction and knowledge regarding their influence during the different times of pediatric age can be a useful guide for the clinical practice and constitutes the main propose of this review.

## 0 to 6 months – exclusive milk diet

The first 6 months of children’s life are characterized by the rapid synthesis of new tissues and by an exclusively milky feeding [4]. Growth is very rapid at this stage: at 6 months the weight of the birth is usually already doubled, with a length increase of 15 cm and head circumference increase of 8 cm [5].

After a physiological weight loss in the first days caused by a loss of extracellular fluids, growth speed reaches its maximum in the first month (about 180 g/week), with a subsequent decrease in the second month (about 140 g/week). It gradually decreases during the successive months [6]. Besides this, the infant quickly develops new neurocognitive and motor functions [7].

Nutritional assessment in the first 6 months requires a strict observation of feeding and a determination of length, weight and head circumference [8]. Weight and length normal increases are signs of good health; in addition to this, the increase of head circumference reflects the increase of brain mass, which usually reaches the double of its volume during the end of the first year of life [9].

In the first 6 months it is recommended to weigh the infant every week, in order to assess whether the feeding is sufficient to ensure a proper growth [10]. A complete anthropometric assessment should be done at birth, at 1 month of life and every 3 months. The growth curves of the World Health Organization (WHO) represent an essential tool for a proper assessment of growth [5]. A weight > 97° centile means overfeeding, while a weight < 3° centile or a relevant deflection of the growth curve may indicate inadequate nutrition or underlying disease. In these cases, a medical assessment followed by an adequate nutritional intervention is required [11].

### Breast milk

Breast milk is the main food for infant and, during the first 6 months, it meets the whole nutritional needs in terms of energy, proteins, carbohydrates, lipids, vitamins and liquids [12].

WHO recommends an exclusive breastfeeding for the first 4–6 months of life, with a subsequent introduction of complementary foods up to the second year of life [13]. In cases of an exclusive breastfeeding, the average milk production in the first half is around 800 ml/day [12]. Breast milk has an average energy content of 0.67 Kcal/g, containing 8–10 g/L of protein and essential amino acids (Table 1) [12, 14]. Breast milk’s average lipid content is 4 g/L. Within lipids, the 33–38% is made of oleic acid, while linoleic acid corresponds to 8–12%. Docosahexaenoic acid (DHA) is present in variable quantities depending on maternal dietary habits. The average cholesterol content is 160 mg/L. Lactose represents the prevalent carbohydrate of breast milk (70 g/L) [15–17].

**Table 1:** Human milk composition during the first year of breastfeeding [12]

Age (months)	Energy *(kcal/g)*	Protein *(g/l)*	Vitamin A *(μmo/l)*	Vitamin D *(ng/l)*	Vitamin B6 *(mg/l)*	Calcium *(mg/l)*	Iron *(mg/l)*	Zinc *(mg/l)*
**1**	**0.67**	**11**	**1.7**	**645**	**0.13**	**266**	**0.5**	**2.1**
**2**	**0.67**	**9**	**1.7**	**645**	**0.13**	**259**	**0.4**	**2**
**3**	**0.67**	**9**	**1.7**	**645**	**0.13**	**253**	**0.4**	**1.5**
**4**	**0.67**	**8**	**1.7**	**645**	**0.13**	**247**	**0.35**	**1.2**
**5**	**0.67**	**8**	**1.7**	**645**	**0.13**	**241**	**0.35**	**1**
**6**	**0.67**	**8**	**1.7**	**645**	**0.13**	**234**	**0.3**	**1**
**7**	**0.67**	**8**	**1.7**	**645**	**0.13**	**228**	**0.3**	**0.75**
**8**	**0.67**	**8**	**1.7**	**645**	**0.13**	**222**	**0.3**	**0.75**
**9**	**0.67**	**8**	**1.7**	**645**	**0.13**	**215**	**0.3**	**0.75**
**10**	**0.67**	**8**	**1.7**	**645**	**0.13**	**209**	**0.3**	**0.5**
**11**	**0.67**	**8**	**1.7**	**645**	**0.13**	**203**	**0.3**	**0.5**
**12**	**0.67**	**8**	**1.7**	**645**	**0.13**	**197**	**0.3**	**0.5**

Regarding micronutrients, breast milk principal contains minerals such as calcium and phosphorus, and can be considered relatively rich in vitamins A, C and E. The contents of vitamins B1, B6 and B12 can vary according to maternal nutrition [18].

Breast milk superiority in promoting healthy and adequate growth is demonstrated [16, 19], and all practices that encourage breastfeeding are recommended. One of the main practices used with this purpose is the early skin-to-skin contact, which should be performed within the first hour of life [20].

### Energy requirements

Energy needs depend on basal metabolic rate (BMR), growth processes, diet-induced thermogenesis (DIT) and physical activity level (PAL) [21]. However, in the 0–6 month period, the main determinats of energy needs can be considered BMR and growth processes, respectively [22, 23]. In this period, a volumetric increase of organs take place, with a consequent maturation and development of new functions. These changes firstly have an energy cost related to the deposition of new tissues and, secondly, an energy cost linked to the increase in BMR [22].

The direct determination of BMR in infants is not an easy task; for this reason, the evaluation of the resting energy expenditure (REE) is commonly performed by applying the Schoefield formula [24]. The weight increase requires around the 30% of total energy expenditure during the first trimester, and around 8% in the second one [25–27]. The total energy intake from exclusive breastfeeding should be enough to match the energy needs of the first 6 months of life.

### Protein requirement

The increase in weight, length and head circumference of the first semester requires proteins. Their needs vary according to the specific age and reach a population reference intake (PRI) of 1.31 g/kg/day at 6 months [28]. PRI is commonly defined as the level of nutrient intake, which is adequate for virtually all people in a population group, meeting the requirements of at least 97.5% of individuals in the population observed [29]. Adequate intake (AI) is defined instead as the value estimated when a PRI cannot be established; it is usually average observed or experimentally determined among healthy people, but its practical implications and usage are not different from PRI ones [29].

Exclusive breastfeeding allows the infant to meet the protein needs of the first semester, both in quantitative and qualitative terms, by providing essential and conditionally essential amino acids. Protein-energy malnutrition causes growth slowdown and, in severe cases, it can cause a deficiency of the immune function with a consequent predisposition to infections.

Most infant formulas are made with cow’s milk proteins and, even if giving right balance of nutrients to the infant, may contain higher levels of protein than breast milk [4] and in fixed amounts; on the other hand protein levels in breast milk change instead week by week, allowing the milk to progressively meet infants’ needs, which are higher in the newborn. Indeed, breast milk at the third month contains approximately half of proteins (0.8–1.0 g/100 mL) compared to the same milk in the first weeks of life (1.4–1.6 g/100 mL), mostly accounted for by functional proteins with immune activities [4]. High protein formula, instead, in order to ensure the needs of all infants during first months of life and compensate the lower biological quality of cow’s protein component, could result in forms of proteins overfeeding.

An unnecessary consumption of high protein formula milks during the first 2 years of life has been associated to later obesity and overweight in pediatric age [30]. Nevertheless, there is not an unambiguous consensus yet on the optimal protein percentage in infant formulas composition, and scientific societies only define minimum levels (1.8 g/100 kcal), and, on the other side, very high upper limit levels (4.5 g/100 kcal), with no clear evidence on the benefits of reducing protein concentration in infant formulas in terms of long-term outcomes [31].

### Lipid requirement

Lipids have both a plastic and energetic function in the first half of life, containing essential nutrients such as polyunsaturated fatty acids (LC PUFA) and their conditionally essential longer chain derivatives, in particular arachidonic acid (ARA), and DHA; they also allow the absorption of fat-soluble vitamins [32]. These nutrients contribute to modulate the inflammatory response, regulate gene expression and influence the fluidity of cell membranes of various tissues, including those having a high functional specialization, such as brain and retina [33].

Indeed, DHA plays a leading role in the neurologic development of the first months. The lack of the conditionally essential ARA and DHA can lead to functional alterations at the neurological, immunological and cardiological level [34, 35]. Exclusive breastfeeding allows to meet the lipid needs of the first semester both in quantitative and qualitative terms, by directly supplying together the two essential fatty acids (linoleic and alpha-linoleic acid) together with ARA, DHA, and preformed cholesterol, contributing to deliver fatty acids to membranes and target organs [1].

In infants with cholestasis, intestinal lymphangiectasia or other pathologies that determine lipid malabsorption, growth retardation and clinical manifestations related to the deficiency of fat-soluble vitamins (vitamins A, D, E and K) occur since the first months of life [36].

### Carbohydrate requirements

Human milk provides a constant supply of carbohydrates to the infant, allowing appropriate nutrition, maturation and growth rate. Lactose represents the principal carbohydrate and his concentration is 60–78 g/L. Lactose indeed constitutes the 7% of human breast milk composition, and it alone provides the 40% of the total milk energy amount [4]. Even if variable, higher lactose concentrations have been found in the milk of mothers producing higher quantities of milk [16].

A positive association between infant growth rate and volume of milk consumed has been found, with a significant relation between the specific milk total carbohydrates amount and infant length, weight and fat-free mass. Furthermore, lactose and carbohydrates intakes have been shown to be lower in breastfed infants at 3 and 6 months compared to formula-fed infants [37].

Human milk carbohydrates also include small amounts of monosaccharides, such as glucose and galactose, as well as human milk oligosaccharides (HMO). Nearly 200 unique oligosaccharides structures have been identified. HMO composition varies between mothers and during the course of lactation [37]. HMO, a heterogeneous group of complex indigestible sugars, are a very important constituent of human milk. They are considered as “prebiotic” agents that enhance the growth of beneficial probiotic organisms and that can also operate as pathogen inhibitors in the human gut [38].

### Fluid requirement

Water requirements and body water content are higher in infants than in later ages [39]. According to the WHO, the need for liquids in the first semester should be met exclusively by breast milk, avoiding the use of sugary drinks or herbal teas [1, 40].

### Micronutrients

The infant needs micronutrients to support his physiological growth. Breast milk is adequate for infant’s nutritional needs of most micronutrients in the first semester of life: it is rich in minerals and vitamins. Breast milk is relatively deficient in iron (Fe) and zinc (Zn), but, while on one hand they are very efficiently absorbed, on the other their deposits formed during pregnancy may support physiologic needs in the first 6 months of life, up to the beginning of weaning. If an exogenous source of iron is not provided during the second half of infancy, exclusively breastfed infants are at risk of becoming iron deficient [12]. Among minerals, calcium (Ca) and phosphorus (P) are essential for growth and breast milk is indeed rich of them [41]. Iodine is essential for fetus and infant’s growth, its early deficiency causes hypothyroidism and cretinism [1, 25]. Breast milk supplies adequate iodine content to the infant if the mother introduces with diet about 250 μg/day of this mineral [41]. The intake of artificially enriched food, such as salt, allows achieving iodine requirement [20]. Vitamin D is essential for the calcium absorption and bone tissue synthesis. Because breast milk is poor in vitamin D, daily supplementation of 400 IU is recommended in the first year of life [1, 25, 42]. Vitamin A is essential for cell differentiation and modulation of apoptosis [43]. It also regulates embryogenesis, growth, immune function and vision. Zn is involved in numerous cellular functions and is critical for growth and development. It plays a central role in cell differentiation, especially in tissues that are characterized by rapid turnover and proliferation, such as the immune cellular network and the gastrointestinal system [44]. Iron is essential for hemoglobin and new tissues synthesis; it contributes to immune system functioning and nervous system health. Infants in the first months of life may also rely on iron deposits acquired by the mother in utero. In preterms, iron reservoir may be not sufficient, therefore iron supplementation (2 mg/Kg) is recommended in the 1–12 months interval [45, 46]. Some procedures, such as delayed cord clamping, contributes to increase iron deposits in the newborn, reducing the risk of iron deficiency in later ages [20]. Vitamin K allows the activation of coagulation factors and its deficiency determines hemorrhages [47]. Breast milk is deficient in vitamin K, so all breastfed infants should be supplemented with intramuscular administration at birth [48, 49].

## Early childhood (6 months to 2 years)

The growth rate of the second semester of life is lower compared to the first one. Approaching the first year of life, the weight triples, reaching on average 9.5 kg, and the length increases by 25% [5]. At 2 years the weight reaches about 12 kg in males and 11.5 kg in females, with an average increase of the 25% during the second year [5]. Length increases on average by 15% in both sexes. Regarding head circumference, after a rapid increase of the first year, during the second one it only increases by 4 [5].

From month 12 to 24 body composition changes, and the infant reduces his subcutaneous tissue. In addition to the increase in weight and length, all organs mature, and the infant develops new neuro-cognitive and motor functions. During this of period the child is commonly called “toddler”.

After the sixth month of life, breast milk is not sufficient to meet nutritional needs, and the introduction of complementary foods should begin. Weaning is a delicate moment that should be led by the pediatrician to avoid the onset of bad eating habits, the most common of which are unbalanced diets, selective diets, hypo-nutrition and hyper-nutrition. The pediatrician should be able to advise parents about the more appropriate introduction of new foods and the management of problems such as neophobia or the avoidance restrictive disorder of food intake (ARFID). Furthermore, with weaning, food-related diseases such as celiac disease and food allergies can occur, having possible significant repercussions on growth.

Complementary solid foods, including allergenic ones, should be introduced into diet after 4 months, or around the sixth month. No scientific evidence justifies a delayed introduction of solid foods after 6 months of age in order to prevent allergies in infants, with no distinction in high or low-risk ones [50]. In addition to this, several studies have been published demonstrating that delayed exposure to allergenic foods did not reduce the risk of food allergies [51].

The nutritional assessment during early childhood includes the measurement of length, weight and head circumference. Growth is not always constant, so slower growth phases are to be expected, followed by periods in which growth is faster. For this reason, during the first 4 months of life infants should be weighed every week, from 4 to 6 months every month and, in the second semester, every 2 months. Toddlers should be weighed every 3 months.

The data obtained should be interpreted according to WHO growth curves [5], an indispensable tool for the pediatrician evaluating the progressive assessment of growth. A weight > 97th centile indicates overfeeding. A weight < 3rd centile or a deflection of the growth curve indicate inadequate nutrition or underlying pathologies. In both cases, after a necessary exclusion of other disorders, a nutritional intervention is required.

### Energy requirement

In the interval 6–24 months, energy needs depend on BMR, PAL and growth (4,5,6). The energy intake depends on the introduction of proteins, lipids and carbohydrates, which should be taken in a balanced way. Weaning increases the risk of bad eating habits which can lead to low-calorie, high-calorie or unbalanced diets.

### Protein requirement

Protein dietary intake is necessary to meet growth needs. Protein requirement vary according to age: at 1 year of life, the European Food Safety Authority (EFSA) suggests a PRI of 1.14 g/Kg/day [1]. After the first year, the growth is much less rapid, and protein needs decrease. The recommended protein intake is measured as a percentage of the daily energy intake (%E). Up to 2 years of age, the optimal protein intake according to recommended nutrient intake levels (LARNs) is < 15%E, preferably in the 8–12%E range [25].

Protein-energy malnutrition can damage some human tissues, such as brain, immune system and intestinal mucosa. On the other hand, an excessive protein intake (> 15%E) seems to be related to the risk of being overweight or obese in adulthood, which may also increase the risk of developing chronic diseases [52]. This statement is based on multiple evidences that observed how a high protein intake during early childhood and pre-scholar age was associated to an increased BMI, especially in 4–8 years children [30, 53–55]. For this reason, a suggested a maximum level of protein equal to 14–15%E has been suggested by several authors to be observed in early childhood [56, 57]. An useful approach to decrease protein intake could be the promotion of breastfeeding throughout the first year of life, and, after weaning, the avoidance of protein rich foods and cow’s milk excessive consumption [52].

### Lipid requirement

Lipids are the macronutrients with higher calorie density. They allow the absorption of fat-soluble vitamins and are a source of essential fatty acids, ARA, EPA, DHA and cholesterol. The intake of lipids is essential in the first 2 years to support brain growth and development [58]. In early childhood, the quality of fats consumed is more important than quantity. In early childhood, fats should constitute 40%E (Table 2), divided as follows [1, 25]:

- Monounsaturated fatty acids (MUFA) equal to 15%E, mainly represented by oleic acid.
- Saturated fatty acids (SFA) < 10%E.
- Omega 6 PUFA in the range between 4 and 8%E, mainly represented by linoleic acid.
- Omega 3 PUFA in the range 0.5–2%E, mainly represented by alpha-linolenic acid.
- Trans fatty acids < 1%E.
- DHA 100 mg/day.

**Table 2:** PRIs for total fat and carbohydrates and AIs for fatty acids [59]

Age group (years)	Total carbohydrates (%E)^a^	Total fat (%E)^a^	LA (%E)^b^	ALA (%E)^b^	ARA + DHA (mg/day)^b^	DHA (mg/day)^b^
7–11_**(c)**_		40_(b)_	4	0.5		100
1	45–60	35–40	4	0.5		100
2–3	45–60	35–40	4	0.5	250	
4–6	45–60	20–35	4	0.5	250	
7–10	45–60	20–35	4	0.5	250	
11–14	45–60	20–35	4	0.5	250	
15–17	45–60	20–35	4	0.5	250	
≥ 18	45–60	20–35	4	0.5	250	

*ALA* α-linolenic acid, *d* day, *DHA* docosahexaenoic acid, *%E* percentage of energy intake, *ARA arachidonic acid*, *LA* linoleic acid

^a^*PRIs* Population reference intake ranges

^b^*AI* Adequate intake

^c^i.e., the second half of the first year of life (from the beginning of the 7th month to the 1st birthday)

Oleic acid is the main MUFA in the diet, it is found abundantly in olive oil whose intake allows to meet the total lipid requirement avoiding excessive consumption of saturated fatty acids. Among PUFAs, linoleic acid is the precursor to ARA, while alpha-linolenic acid is a precursor to DHA. ARA is essential for infant growth and development and its deficiency can cause dermatitis. ARA is not an essential fatty acid despite its important role: probably for this reason EFSA does not set any dietary reference value (DRV) for ARA [1]. ARA is always present in human milk and is added in infant formulas, but during late infancy, the amount of dietary ARA provided by solid food is low [60]. DHA is essential for the development of the heart, brain and retina, and its lack could provoke adverse outcomes on psychomotor and visual development [61]. Since the endogenous production of DHA cannot cover the needs, the child should add an adequate intake of 100 mg/day by natural food [1, 25]. Cold sea fish (cod, salmon, eels) or some algae contain a high amount of DHA. Cholesterol is a precursor to steroid hormones, bile acids and vitamin D; it also participates in the formation of cell membranes. Principal foods containing cholesterol are milk, eggs and meats [62].

Contrary to what it has been observed with the excessive intake of protein in early childhood, high fat intake in the same period seems to have no effect on inducing overweight and obesity, measured with indexes of adiposity in later ages [63].

### Carbohydrate requirements

Carbohydrates are macronutrients with an exclusively energetic function (4 Kcal/g) [64]. They are indispensable for the metabolism of erythrocytes and vital organs such as kidney and brain. There is a reference interval for carbohydrate intake related to early childhood. The minimum limit of 45%E [1, 25], covers the metabolic needs of glucose in the tissues and allows to contain the total lipid intake of the diet by 40%E and that of proteins within 15%E [25]. The upper limit of the reference range is 60%E [1, 25]. Carbohydrates are divided into simple sugars, oligosaccharides and polysaccharides. Simple sugars can be naturally present in foods or added; they are not indispensable if the total carbohydrate requirement is satisfied with other available carbohydrates (polysaccharides, starches) [1]. Their excessive intake can cause hyperglycemia, steatosis, overweight, obesity, diabetes, dyslipidemia, hypertension and dental caries. In early childhood, LARNs recommends an intake of simple sugars < 15%E [25], while ESPHGAN recommends an intake < 5%E [65].

### Fluid requirement

In early childhood, water represents the 70% of body composition. From fourth to sixth month, with weaning, foods introduced have lower liquid content than it has been in the first semester; therefore, water becomes indispensable to meet the need for liquids. The adequate fluid intake is 800 ml/day in the second semester of life and rises up to 1200 ml/day in the second year [1].

### Micronutrients

During early childhood, minerals and vitamins are essential for growth. After weaning, a varied diet is essential to obtain adequate micronutrient intake. Calcium, phosphorus and vitamin D are essential for bone growth; iodine allows thyroid hormones synthesis and brain myelination; iron is mainly necessary for the synthesis of red blood cells and new tissues. Finally, zinc is essential for the growth and regulation of the immune system. Signs of various micronutrients deficiencies in infants and children who needs parenteral feeding, and their recommended doses, are described in Table 3.

**Table 3:** Signs of micronutrients deficit in infants and children parentally fed and recommended doses for parenteral supply in the same population [66, 67]

	Signs of deficiency	Infants	Children and adolescents
Vitamin A	night blindness	150–300 μg/Kg/die	150 μg/die
Vitamin D	ricket	400 IU/die	400–600 IU/die
Vitamin E	cholestasis, liver damage	2,8–3,5 mg/die	11 mg/die
Vitamin K	bleeding	10 μg/Kg/die	200 μg/die
Vitamin C	mucosal bleeding, scurvy	10–25 mg/Kg/die	80 mg/die
Thiamine B1	Beri-beri, lactic acidosis, Wernicke’s encephalopathy	0,35–0,5 mg/Kg/die	1,2 mg/die
Riboflavin	mucosal hyperemia, stomatitis, dermatitis, anemia, eye disorders	0,15–0,2 mg/Kg/die	1,4 mg/die
Pyridoxine B6	dermatitis, seizures, hyperhomocysteinemia, anemia, depression, encephalopathy	0,15–0,2 mg/Kg/die	1 mg/Kg/die
Niacin	Pellagra (skin, intestinal and neurological problems)	4–6.8 mg/Kg/die	17 mg/die
Vitamin B12	megaloblastic anemia, neurological disorders	0,3 μg/Kg/die	1 μg/die
Biotin B8	lethargy, hypotonia, irritability, alopecia, dermatitis, anorexia, pallor, glossitis, nausea, hyperesthesia, muscle pain, hypercholesterolemia	5–8 μg/Kg/die	20 μg/die
Folic acid	hyperhomocysteinemia, megaloblastic anemia	56 μg/Kg/die	140 μg/die
Iron	anemia	50–100 μg/Kg/die	50–100 μg/Kg/die
Zinc	stunted growth, infections, typical skin rash	100–250 μg/Kg/die	50 μg/Kg/die
Iodine	impaired thyroid function	1 μg/Kg/die	1 μg/Kg/die
Selenium	erythrocyte macrocytosis, depigmentation, muscle weakness	2–3 μg/Kg/die	2–3 μg/Kg/die

### Calcium

Calcium needs depends on the development of bone mass. Ca is mainly contained in milk, cheese, yoghurt and vegetables. Calcium deficiency in pre-adolescent age causes rickets. In the 6–12 months interval, the adequate intake is 280 mg/day, during the second year, the recommended intake for the population increases to 450 mg/day [1].

### Phosphorus

Phosphorus is widely contained in foods, particularly in cereals, wholemeal flours, eggs, legumes, fish, milk, cheeses and meat. Given the wide distribution of phosphorus in food, deficiencies linked to insufficient food intake are rare. Chronically insufficient intake can compromise growth and cause rickets. From 6 to 12 months, the AI of phosphorus is 160 mg/day, while in the toddler, the recommended intake for the population increases to 250 mg/day [1, 25].

### Iodine

Iodine is essential for thyroid hormones synthesis. Its deficiency in early childhood can cause goitre and hypothyroidism [68]. Most of the foods are deficient in iodine, but their fortification allows them to reach the age-appropriate intake level, which is 90 μg/day [1].

### Vitamin D

Vitamin D is essential for calcium absorption and bone tissue synthesis. Its receptor is expressed in different cells, and for this reason vitamin D plays its role in many nonskeletal mechanisms. Several studies support a relationship between serum 25-hydroxyvitamin D [25-(OH)D] levels and chronic metabolic, cardiovascular, and neoplastic diseases. However, vitamin D assumption is not recommended to prevent and treat chronic nonskeletal disorders in this phase of life [69].

The skin can produce vitamin D after solar exposure, but the child can also introduce it with food. Cod liver oil, fish, pork liver, eggs and butter naturally contain D vitamin. Vitamin D deficiency in early childhood causes rickets. Endocrine Society Clinical Practice Guidelines recommends using the serum circulating 25-(OH) D level to evaluate vitamin D status in patients who are at risk for vitamin D deficiency. Institute of Medicine (IOM) and Endocrine societies agree that there is no need to screen the general population routinely [70]. Endocrine society define vitamin D deficiency as a 25(OH) D below 20 ng/ml, and vitamin D insufficiency as a 25(OH) D of 21–29 ng/ml [70]. IOM asserts instead that the 20 ng/ml level is the upper range of human requirements [71].

The available evidence on serum 25(OH) D concentration and musculoskeletal health outcomes is suitable to set vitamin D intake for healthy infants and children. A serum 25(OH) D concentration of 20 ng/ml is a suitable target value to set the DRVs for vitamin D [1]. Supplementation in infants is essential to ensure healthy bone growth and prevent rickets. In infants (0–12 months) the AI is 10 μg/day, in the it toddler rises to 15 μg/day [1, 25, 70, 71]. Doses 100 times higher than the recommended daily intake can cause toxicity phenomena [72].

### Vitamin A

Vitamin A is contained in the liver of several animal species, as well as in eggs and fish. Vegetables contain carotenoids, a pro vitamin from which vitamin A derives. Retinol equivalent (RE) is the unit of measure used to describe the total quantity of vitamin A (vitamin and pro vitamin) in the diet. In developing countries, at high risk of malnutrition, vitamin A deficiency can cause night blindness. The lack of vitamin A, in some cases, causes conjunctival lesions, xerophthalmia or keratomalacia [73]. This condition, if untreated, progresses until the destruction of the anterior chamber of the eye, which can lead to irreversible blindness. Chronic over administration of vitamin A causes liver damage, reversible after discontinuation of supplementation. In the second half of the year, the AI is 250 μg RE/day [1], while in the child, the recommended intake for the population is 300 μg RE/day [1, 25].

### Zinc

Zn is essential for growth, immune and gastrointestinal systems. Infant and toddler have an increased risk of Zn deficiency, a condition characterized by growth retardation, alopecia, diarrhea, skin lesions, loss of appetite and reduced antioxidant defenses [74–76]. Another effect of Zn deficiency is impaired immune function [77]. Excessive intake of Zn supplements can cause intestinal disorders and fever. The recommended Zn requirement for the population in young infant is 2,9 mg/day and rise up to 4,3 mg/day in the toddler [1].

### Iron

The infant faces the first months of life with iron deposits acquired in utero, but after the sixth month dietary intake becomes essential. Meat, fish, cereals and eggs contain iron [25, 78]. Among tissues, bone marrow requires large quantities of iron for the synthesis of hemoglobin. Iron deficiency manifests itself with reduced physical performance due to the reduction of hemoglobin and myoglobin [79]. Other consequences of the deficiency state are the impairment of the immune response with a reduction in the function of macrophages and neutrophils and a decrease of T lymphocytes [80]. Brain iron deficiency causes reduced myelin and neurotransmitter synthesis with impaired movement, memory and perception control [81]. Infants iron deficiency can cause irreversible brain damage. In the 6–12 months interval, the recommended requirement of Fe for the population is 11 mg/day [1]. From the beginning of the second year, it decreases to 7 mg/day [1, 25]. Supplementation is only recommended in overt cases of iron deficiency.

### Vitamin K

Vitamin K activates coagulation factors. Its severe deficiency causes bleeding. From weaning, the infant can acquire this vitamin through the diet. The foods that contain it most are green leafy vegetables, such as broccoli, lettuce and spinach. The AI in the 6–12 months interval is 10 μg/day [1, 25]; in the toddler, it increases to 12 μg/day [1].

### Vitamin C (ascorbic acid)

Vitamin C participates in enzymatic reactions such as biosynthesis of collagen, carnitine and norepinephrine. It also works in non-enzymatic reactions, such as neutralization of reactive oxygen species [82]. Vitamin C is found mainly in vegetables. Its deficiency is usually due to an inadequate intake and causes scurvy. This disease manifests itself with fatigue, weight loss, arthralgias and gums bleedings; subsequently, if left untreated, it induces the formation of hematomas (especially in the lower limbs). It also causes impaired scarring, tooth loss, bruising and bleeding in many organs, sometimes with a fatal outcome. In the infant and toddlers, the AI for vitamin C is 20 mg/day [1].

### B vitamins

B vitamins are water-soluble nutrients involved in different metabolic processes, allowing the use of carbohydrates, lipids and proteins to obtain energy. They are fundamental for the development of all organs and systems, particularly the nervous system. Humans cannot synthesize B vitamins and need to introduce these nutrients with food. The infant takes these vitamins from breast milk, subsequently, from the moment of weaning, the intake of the B complex vitamins depends on other foods. A diet rich in whole grains, green leafy vegetables, oilseeds, dried fruit and legumes ensures a sufficient dose of B vitamins. A deficiency in any vitamin of the B complex is often followed bay lacking of other vitamins of the same group [83]. For this reason, it is more practical to supplement the diet with the full group B.

### Thiamine (B1)

Thiamine acts as a coenzyme in the metabolism of carbohydrates, branched-chain amino acids, fatty acids and nucleotides [84]. It allows the synthesis of acetylcholine and also act as a neuromodulator. For these reasons, it may be qualified as essential for the central and peripheral nervous system. A severe and protracted thiamine deficiency causes beriberi, a disease characterized by neurological, cardiovascular and gastroenteric manifestations.

### Riboflavin (B2)

Riboflavin participates in oxidation-reduction reactions in metabolic pathways involving carbohydrates, lipids and amino acids [85]. Riboflavin deficiency causes skin lesions, anemia, neuropathy and corneal vascularization.

### Niacin (B3)

Niacin is the constituent of enzymatic cofactors that participate in many metabolic reactions of oxidation-reduction. It contributes to the metabolism of fats, carbohydrates and proteins. It helps the nervous system in its functions, improves circulation and reduces blood cholesterol. Severe niacin deficiency leads to pellagra, a disease characterized by dermatitis, diarrhea and dementia [86].

### Pyridoxine (B6)

Pyridoxine is a cofactor in the folate cycle, and the synthesis of neurotransmitters such as dopamine, serotonin, GABA, norepinephrine and melatonin. B6 deficiency can lead to reduced production of these neurotransmitters and, consequently, to sleep and behavior disorders, cognitive decline, peripheral neuritis, cardiovascular diseases, anemia and dermatitis [87].

### Biotin (B8)

Biotin is the coenzyme of some carboxylases involved in energy metabolism, in the synthesis of fatty acids and the catabolism of amino acids. A primary biotin deficiency is rare. Biotin deficiency can occur in cases of protracted parenteral nutrition (Table 3) and can manifest itself with eczema, mood alteration and immunodeficiency [88].

### Folic acid (B9)

Folic acid is necessary for DNA synthesis, repair and methylation reactions. Therefore, it allows cell division reactions in case of rapid growth and also enables the metabolism of homocysteine. Humans cannot produce folic acid, so they find this vitamin in foods such as green leafy vegetables, legumes, cereals, fruit and liver. Folic acid deficiency causes anemia and an increase in serum homocysteine. In the 6–12 months interval, the AI for folic acid is 80 μg/day, while the recommended requirement for toddlers rises to 120 μg/day [1].

### Vitamin B12

Vitamin B12 participates in the metabolism of homocysteine and allows the synthesis of DNA and hemoglobin.

Food of animal origin, such as sheep liver, veal, chicken and turkey are rich in vitamin B12. Some fish also contain vitamin B12. Eggs and cheeses contain B12 in much lower concentrations than meat and fish. B12 deficiency causes megaloblastic anemia, increased serum homocysteine and can lead to permanent neurological damage [89]. In infant and toddler, the AI of vitamin B12 is 1.5 μg/day [1].

## From preschool age to adolescence

Preschool age is commonly considered the period between the third and sixth year of life. In these years the growth is almost constant: around 2 kg and 6 cm per year. The prominence of the abdomen and the evident lumbar lordosis found in infants and toddlers usually disappear. School-age begins at 6 years and ends with the onset of puberty, which occurs between 9 and 15 years for female subjects, and between 12 and 17 years for male subjects. The average weight increase is 3–3.5 kg per year and about 6 cm in height [5]. Adolescence is a time of notable growth during which a significant growth spurt occurs with an average bone mass increase of 45%. Soft tissues, organs and even red blood cells grow in size [90]. To support all these changes, there is a nutritional requirement peak during adolescence and macronutrient or micronutrient deficits can compromise growth and delay sexual maturation [91].

Regarding nutrition, the diet of the child should be guided by the pediatrician and the family who should propose a varied diet with adequate portions. During puberty, there is usually a change in habits, becoming more exposed to the risk of “bad habits”. In essence, the number of meals “away from home” increases with a preference for “junk food” which is rich in fat and deficient in micronutrients. Growth assessment involves the measurement of height and weight. During adolescence is necessary to integrate the evaluation of the pubertal stage. Other important parameters are height velocity, abdominal circumference and the body mass index (BMI). The abdominal circumference represents a valid marker of cardiovascular risk in subjects with excess weight. BMI describes the relationship between weight and height: it increases in the first year of life, then it decreases up to a nadir and starts to rise again at 5–6 years. The resumption of BMI growth before 5 years is called early adiposity rebound and is an early indicator of the risk of developing obesity [92, 93]. Regular auxological and nutritional assessments allow early detection of overweight, obesity and erroneous lifestyles which, if not corrected in childhood, increase the risk of chronic degenerative diseases such as diabetes and hypertension [94]. Low weight or deflection of the growth curve may be early sign of organic pathologies or eating behavior disorders.

### Energy requirement

From childhood to adolescence, energy intake should be adequate to the needs to avoid malnutrition both in defect or in excess. With puberty onset, the energy requirement rises, reaching 2500 Kcal/day in girls and 3000 Kcal/day in boys [25]. The increase in energy intake should be satisfied with the consumption of cereals, legumes, fruit and vegetables, rather than with the input of fats and free sugars. Teenagers, especially during the pubertal spurt, should avoid low-calorie diets because they can slow down sexual growth and maturation. They also should avoid over-nutrition that promotes overweight and obesity.

Childhood obesity is a serious health emerging concern. Obese children are much more likely to become obese as adults compared to children with a normal BMI. Obesity in childhood can cause serious chronic diseases later on, such as type 2 diabetes, cardiovascular disease, hypertension, osteoporosis and some carcinomas. It also has psychological consequences and can contribute to a delay in academic and social function as well as poor self-esteem and depression. Thus, the prevention of childhood obesity is of primary importance. The interventions for preventing and controlling obesity are mainly aimed at limiting the intake of sugar and high calorie snacks with higher consumption of vegetable- and fruit-based diet. It is important to have daily healthy breakfasts and home cooked family meals, adequate portion size, and a reduction in eating-out. Also limiting the duration of “screen time” and increasing the level of physical activity can prevent childhood obesity [95].

### Protein requirement

In children, dietary protein intake is necessary to replenish protein turnover and to meet growth needs. The daily protein requirement from 4 to 7 years is 0.86 g/kg (PRI) and rises to 0.92 g/kg in the pre-pubertal phase [1, 28]. The adolescent’s higher protein requirement is necessary to support the increase in muscle mass, erythrocytes and myoglobin and to support hormonal changes. Insufficient protein intake causes delayed growth and sexual maturation, reduced muscle mass and immunodeficiency [96]. Food proteins can be of animal or vegetable origin. Compared to plant proteins, animal proteins contain a greater quantity and variety of essential amino acids, and, for this reason, they are considered of higher quality [97].

### Lipid requirement

Lipids are a consistent source of energy, contain essential micronutrients and allow the absorption of fat-soluble vitamins. Still, their excessive intake in childhood increases the risk of chronic degenerative diseases in adulthood. Considering that lipids are essential for brain growth in the first 2 years, pediatricians should give dietary advice to reduce cardiovascular risk from the third year of life [58]. From preschool to adolescence, the recommended lipid requirement for the population is in the range of 20–35%E (Table 2), divided as follows [1, 25]:

- Saturated fatty acids (SFA) < 10%E.
- PUFA omega 6 in the range between 4 and 8%E.
- PUFA omega 3 in the range 0.5–2%E.
- Trans fatty acids < 1%E.
- DHA 100 mg/day;
- Cholesterol < 300 mg/day.

Family’s habits can influence and guide the eating habits of the child. Teenagers, on the other hand, develop disordered eating behaviors by preferring foods that are very rich in saturated fat and cholesterol. These incorrect eating habits, together with a sedentary lifestyle, may contribute to the development of overweight and obesity, steatosis and cardiovascular diseases in later ages [98].

### Carbohydrate requirements

From preschool to adolescence, the reference interval for carbohydrate intake is between 45 and 60% of the total dietary energy. In subjects that carry out intense sports activities, contributions of up to 65%E are allowed [25]. Among carbohydrates, simple sugars should be introduced in moderation because they increase the risk of being overweight, obesity, diabetes, hypertension and tooth decay [1]. For these reasons, the maximum intake allowed by LARNs is 15%E [25], and European Society for Paediatric Gastroenterology Hepatology and Nutrition (ESPGHAN) recommends an intake < 5%E [65]. According to these recommendations, in childhood and adolescence, it is appropriate to reduce the consumption of sugary foods such as sweets, fruit juices and sugary drinks. Polysaccharides are instead contained in foods such as bread, pasta, rice, potatoes. Each of these foods has a particular glycemic index that describes the rate of increase in blood sugar following its intake. LARNs recommend preferring foods with a low glycemic index [25].

### Fibre

Dietary fibre is not an essential nutrient because it does not have a plastic or energy function. However, it regulates intestinal function by influencing the microbiota, increasing the fecal mass and accelerating the intestinal transit time [94]. Whole grains, legumes, fruit and vegetables are naturally fibre-rich foods. These foods are also rich in micronutrients, slow down the absorption of carbohydrates and reduce the absorption of fats. In children, the evidence to establish AIs for dietary fibre is scarce. In children of school age, a minimum fibre intake of 10 g/day is recommended, while in teenagers an intake of at least 25 g/day is recommended [1, 25, 55].

### Fluid requirement

Adults offer to child many types of drinks, but not all of them are necessary or even useful for a healthy growth. In the first 5 years of life, children only need water and natural milk. The first one provides the hydration required to live, and the second one provides essential nutrients. The use of sugary drinks can become a negative habit that begins in childhood and strengthens in adolescence. Sparkling beverages, sports drinks, fruit drinks, lemonade, sugar water and other drinks containing added sugar may be considered a risk condition to a child’s health. They increase the risk of being overweight, heart disease, diabetes and hepatic steatosis [99]. Total fluid intake suggested by EFSA in adolescents corresponds to 2100 for males and 1900 ml for females [100].

### Micronutrients

In preadolescent-age, the need for micronutrients is satisfied with a varied diet that includes cereals, legumes, fruit, vegetables, eggs, milk and derivatives, fish and meat. Children don’t need supplements, but some foods such as salt are enriched with iodine to avoid goitre and hypothyroidism. Furthermore, in order to reduce the risk of hypertension in adulthood, it is recommended to reduce the salt content of diet. During adolescence, with the growth spurt, the requests for micronutrients increase, in particular, calcium, iron, zinc and folate; to ensure such income, teenagers should have a very varied diet.

Due to the rapid bone growth taking place, adolescence is the phase of life with the greatest need for Ca (1150 mg/day) [1]. It is not clear whether Ca deficiency in youth can be recovered in adulthood or whether this deficit exposes the individual to a higher risk of osteoporosis in later ages. If the adolescent does not take adequate calcium in the diet, supplementation is recommended. Excessive intake of Ca reduces the absorption of Fe, Zn, Mg and other minerals [101, 102].

Teenagers also have an increased risk of iron deficiency anemia. Fe is essential for increasing muscle mass and red blood cells. Adolescent girls lose iron monthly due to the menstrual cycle and may have an increased risk of anemia. Teenagers should be investigated for iron deficiency anemia every 2 years to treat anemic ones with iron supplementation. In some cases, iron supplementation does not resolve anemia, due to the high needs or because iron is not always the cause of the anemia itself. Still, other micronutrients are necessary for the formation of red blood cells (pyridoxine, folate, magnesium and zinc) [101, 102]. In adolescent, EFSA recommended need for Fe is 13 mg/day, even if adolescent iron needs are highly variable [1]. For this reason, suggested dose for daily elemental iron supplementation in school-age children and adolescent girls, according to WHO guidelines, is 30–60 mg [103].

Teenagers, due to their speed of growth, have a high requirement of Zn and a greater risk of deficiency of this mineral. The consequences of this deficit are linked to the decrease in its biological functions (catalytic, structural, regulatory and immune), which determine alterations of hormones, cytokines and enzymes involved in various services such as bone development, immune system health, skin integrity and adaptation to the dark. The following signs may occur in adolescents with significant Zn deficiency: growth retardation, hyporexia and hypogonadism. These issues regress after supplementation. Excessive introduction of Zn can reduce the absorption of Fe [102]. The recommended requirement for Zn in adolescent in about 11 mg/day [1].

Many adolescents have an insufficient dietary intake of folate and may develop a deficiency of it, resulting in anemia and an increase in plasma homocysteine. Furthermore, if we consider the phenomenon of teenage pregnancies, a folic acid deficiency is associated with a higher incidence of neural tube defects in the fetus. In light of the previous considerations, the pediatrician should recommend the intake of foods rich in folate, and in some cases may also offer supplementation with supplements [101]. The estimated folate requirement for the adolescent population is 400 μg/day [1, 104], and, in those considering pregnancies, a supply should be started at least 4–8 weeks before pregnancy begins.

The vegan diet is becoming increasingly popular among teenagers. This diet is based on the exclusion of food of animal origin, including milk and eggs. Although the intake of foods such as legumes, tofu, cereals and dried fruit ensures an adequate energy and protein intake, this diet is deficient in vitamin B12 and riboflavin; therefore a supplementation may be necessary [105]. The recommended B12 requirement for the adolescent population is 3,5 μg/day [1].

## Conclusion

An adequate nutrition ensures a physiological growth while preventing diseases. Both macronutrients and micronutrients are essential for children nutrition in proper amounts and balance. The main macronutrients are proteins, carbohydrates and lipids, but also fibers and liquid intake are important. Also, micronutrients are essential: the lack of even one of them can have important consequences, impairing growth, delaying maturation or determining deficiency diseases such as rickets, scorbutus, cretinism. Every age has its specific nutritional special needs. During the first 6 months of life, growth is rapid, and breast milk, in spite of relatively small amounts of some macro and micronutrient, is able to fulfill growth and development in an optimal way. Weaning increases the risk of an unbalanced diet, and pediatricians should support the family during this process. Growth is constant from pre scholar age to adolescence. With puberty onset, a rapid increase in height and weight can be observed, and also the sexual maturation is performed: these changes need a proportional supply of macronutrient and micronutrient, because undernutrition delays sexual maturation, while overfeeding predisposes to obesity, diabetes and cardiovascular diseases. In order to support the physiological processes of different ages, a deep knowledge of specific nutritional needs of the young, and how they change according to his age and health status, is a useful tool of the clinical practice, and should be possessed by the clinician. In fact, each period of the pediatric age has its specific requirements and characteristics, and the deficiency of specific single nutrients could delay growth or compromise specific organs and functions.

## Data Availability

The datasets used and analyzed during the current study are available from the corresponding author on reasonable request.

## Abbreviations

AI: Adequate intake
ARA: Arachidonic acid
ARFID: Avoidance restrictive disorder of food intake
DRVs: Dietary reference values
DHA: Docosahexaenoic *acid*
DIT: Diet induced thermogenesis
EFSA: European Food Safety Authority
EPA: Eicosapentaenoic *acid*
LARNs: Recommended nutrient intake levels
LC-PUFA: Long chain polyunsaturated fatty acids
BMR: Basal metabolic rate
MUFA: Mono unsaturated fatty acids
PRI: Population reference intake
RE: Retinol equivalent
SFA: Saturated fatty acids
WHO: World health organization
%E: Percentage of daily energy intake
